# Foreign Summary

**Published:** 1839-10-05

**Authors:** 


### FOREIGN SUMMARY.

### Velpeau's Clinical Lectures on Ophthalmia. No. III.

*On the Treatment of the various forms of Blepharitis.*

I have hitherto laid aside all consideration of specific causes and constitutional peculiarities, in speaking of this class of diseases. I shall, therefore, follow the same plan now we have to determine the treatment which should be adopted.

If we take a rapid survey of the various therapeutical means we can oppose to each of these affections when they are purely inflammatory, we shall see that they may all be divided into two classes—those which are direct, and those which are indirect.

Having made this division, I must state, that, in my opinion, all indirect remedies, such as blood-letting, baths, revulsives, &c., ought merely to be considered as adjuvants, useful, it is true—necessary even in some cases; but never to be depended upon alone, if you wish to obtain a radical cure. My opinions on this subject have not been formed *à priori;* they have been adopted only after mature consideration, and are founded on a great number of experiments, many of which some of you have witnessed. No doubt the symptoms of the malady are often more or less mitigated by an indirect plan of treatment, which may consequently be of great assistance towards effecting a final cure; but, in the great majority of cases, it does not suffice alone. The affection is local and external, and as such, a local treatment only can arrest its progress, and effectually restore the tissues to their normal state.

We will now examine the treatment of each form of blepharitis in particular. I intend, at the termination of these lectures, to resume the treatment of ophthalmia, taken in a general point of view.

#### *Mucous Blepharitis.*

Topical astringents are the best remedies we can employ against this form of inflammation; and among these, liquid collyria are by far the most efficacious. Numerous astringent collyria have successively enjoyed the favour of the profession; from these we may select three—those containing nitrate of silver, sulphate of zinc, and sulphate of copper; their efficacy being, in my opinion, much more clearly established than that of any other. I have often tried these collyria, and have always derived great advantage from their use. The one which contains the nitrate of silver, appears, however, to produce a more rapid amelioration than the other two; I now seldom make use of any other. It is, indeed, a most valuable remedy, as most of you who have followed my practice for any length of time must acknowledge. The formula which I follow, varies as the inflammation is more or less acute. I generally begin with half a grain or a grain of the nitrate of silver to an ounce of distilled water, increasing progressively to six, eight, or even ten grains to the ounce of water.

This treatment is, you see, extremely simple; nevertheless, for it to succeed, certain precautions must be used, and it is owing to their having been neglected that some practitioners have not derived from the remedy the benefit they had been led to anticipate. This remark may not not only find its application in this instance, but also in many others in the course of these lectures.

The first point to be ascertained is, that the prescription has been accurately prepared. This is of extreme importance where the quantities are so small, and ought especially to be attended to in hospital practice. Two or three drops of the collyrium should then be instilled into the eye, twice or thrice a day, care being taken, at the same time, that the liquid comes in contact with every portion of the inflamed membrane. This may be attained by directing the patient to lean his head back, and then gently separating the eyelids with the fore-finger and the index of one hand, whilst with the other you allow a few drops of the liquid to fall into the eye from the phial itself. The patient should then be directed to turn the eye in different directions for a few seconds. This must be repeated several times in the course of the day, as already stated, for three or four days following, and the strength of the solution increased, should it appear necessary. The patient may then be allowed to rest for a day or two, when the collyrium is again resumed, as before. It often occurs that the amelioration is very slight whilst the remedy is used, but that as soon as it is laid aside the inflammation rapidly abates. This circumstance leads many persons to conclude, that, so far from having been benefited by the collyrium, their cure has been retarded. This, however, is a most erroneous opinion, and one which only a person unacquainted with pathology could form.

Thus treated, this disease seldom lasts more than ten days—that is, when purely inflammatory, without any complication, as we now suppose it to be. In some cases the inflammation will disappear in less than twenty-four hours. I must not omit to say, that if the patient is strong, robust, of a sanguineous temperament—if he complains of cephalalgia, and the face is flushed, it will be well to have recourse to bleeding. Such a measure, however, I must again remind you, is not indicated by the malady itself, but by the general state of the individual.

*Glandular Blepharitis.*

General treatment is not more efficacious in this form of blepharitis than in the one we have just examined. Ointments, however, should be preferred to collyria, as the latter do not remain sufficiently long in contact with the seat of inflammation. The astringent ointments employed against this affection are almost innumerable. Those most generally used are the ointments of Janin,* Lyon,† Regent,‡ Desault,§ and those containing the nitrate|| of silver and the bichloride of mercury.¶ These ointments, which are efficacious preparations, should not be used indiscriminately in every case. If the glandular inflammation assumes the diphterical form, the best application is the white precipitate ointment, the proportions of which are, Axun. ℥i., Hydrar. Bichlor. ℨi. Should the circle or ribbon I described become red and shining, you must have recourse to the ointments of Janin, Lyon, Regent, or Desault. If there are slight ulcerations along the internal free edge of the palpebræ, and the secretion from the Meibomian glands is very abundant, the nitrate of silver ointment must be preferred.

You have had ample opportunity in our wards to convince yourselves, that a treatment directed on these principles is decidedly efficacious. You must, however, always bear in mind, that glandular blepharitis is an extremely obstinate malady, one which will long set at defiance every means we employ. Aware of this circumstance, you must be cautious not to promise your patient more than you afterwards may find yourself able to perform.

*Granular Blepharitis.*

This form of inflammation is extremely difficult to eradicate, more so than any we have yet spoken of, as you may learn by examining several patients now in our wards. You will find that they have been for some time affected with this complaint, and that every means which has been hitherto employed has failed. I have myself occasionally been successful, but never until after a lengthened treatment. If you keep in mind the extreme tenacity of this complaint, you will not be surprised to hear that the remedies which have been recommended or employed are exceedingly numerous. These remedies are principally astringent topical applications; their number, indeed, is so great, that I shall not even attempt to name them, but merely lay before you the results of the frequent trials I have made of the more important preparations.

When I first began to treat these diseases, I thought the collyrium containing the sulphate of zinc likely to prove beneficial. I soon found, however, that no reliance could be placed upon it, and have, consequently, entirely laid it aside. I next had recourse to a rather strong solution of the nitrate of silver, eight or ten grains to an ounce of distilled water. The greater number of you have seen how slight is the benefit to be derived from this remedy. Finding that these collyria appeared to have little or no influence, although so efficacious in some forms of ophthalmia, I successively tried all the principal preparations recommended by authors. Thus, in a great number of cases, I employed collyria containing subacetate* of lead, deutochloride† of mercury, calomel.‡ I also employed laudanum in every form, the collyrium of Dupuytren,§ the ointments of Janin, Desault, Regent, &c., oxide of bismuth pulverized, a powder formed with equal parts of calomel and sugar: none of these remedies, however, appeared to modify the disease. I then had recourse to caustics. In 1831 I had under my care a female, who had been labouring under very severe granular blepharitis for some months. The conjunctiva of the superior eyelid—the principal seat of the disease—was thickened and covered with fungous granulations. I cauterized lightly, five or six times, the inflamed surface with the solid nitrate of silver, at six or eight days' interval, and obtained a complete cure. Since that time the nitrate of silver, although sometimes successful in my hands, has often failed to produce a similar result. Should you make use of this remedy, you must be careful how you apply it. The nitrate of silver should be drawn slightly across the inflamed conjunctiva, merely so as to whiten the surface. Were the cauterization to give rise to loss of substance, a cicatrix would be formed, which would in all probability produce entropium, with the usual train of disagreeable symptoms. When the solid nitrate of silver is used, this accident may occur, although the greatest circumspection has been employed; I have, therefore, sometimes substituted for it the sulphates of copper or of iron, the action of these preparations not being quite so energetic. The use of these remedies has been attended with advantage to the patient in some instances, but generally the disease has not been perceptibly modified.

* The following are the formulæ of these preparations:

℞. Adipis, oz. ss.; Tuthiæ—Bol. Armen. aa. 2 drachms; Hydrar. Bichlor. 1 drachm. Misce.

† ℞. Hydrar. Binoxyd. gr. xv.; Ung. Rosati, 4 drachms. Tere et misce.

‡ ℞. Buthyri Hydrolato Rosarum Loti, 18 drachms; Camphoræ gr. vi.; Oxidi Hydrar. 1 drachm; Acet. Plumbi Cristal. 1 drachm. Tere simul Oxidum Hydrargyri et Acetatem Plumbi, adde Camphoram ante pulverisatam, dein buthyrum.

§ ℞. Hydrar. Binoxyd. Tuthiæ prepar., Acet. Plumbi, Alum. usti, aa. 1 drachm; Hydrar. Bichl. gr. xii.; Ung. Rosati, 1 ounce. Misce.

|| ℞. Adipis, 1 drachm; Nitra. Argen. gr. i. Misce.

¶ ℞. Hydrar. Bichlor. 1 drachm; Adipis, 1 ounce. Misce.

* ℞. Subacet. Plum. gr. v. ad xv.; Aquæ destill. oz. iv. Misce. Ft. collyr.

† ℞. Hydrar. Bichlor. gr. ½; Aquæ destill. oz. i. Misce.

‡ ℞. Calomel. dr. i.; Aq. destill. oz. vi. Misce. Ft. collyr.

§ ℞. Sacchari Albi, drachm ii.; Hydrarg. Binoxy. gr. x.; Tuthiæ, gr. xx. Tere et misce.

I have not only given a trial to every kind of topical application likely to prove beneficial, but I have also had recourse to the various direct methods of treatment recommended by ophthalmologists. In a great number of cases I have applied blisters to the nape of the neck, the temples, the mastoid region, the arms, and the legs; I have even applied them over the orbit itself. In no instance, however, did these measures seem to make much impression on the disease. I have also tried blood-letting, colchicum, purgatives of all kinds—mercurial, saline, resinous—and still the results have been as unsatisfactory as before. I have employed iodine internally and externally, in every shape, and in a few instances with apparent success. The patients, however, were of a lymphatic constitution, had been long affected with this form of blepharitis, and had been treated in every possible manner. I dare not, therefore, assert that the iodine effected the cure; it may have done so, or it may not. The remedy which I have perhaps found the most efficacious, is a powder containing sulphur and calomel; and yet, although in many cases it has proved exceedingly beneficial, its efficacy, I must confess, has in other instances often appeared doubtful.

This survey of the various remedies employed in the treatment of granular blepharitis has not led us to any very satisfactory conclusion; indeed, a really efficacious treatment of this disease is yet to be found. In the present state of our knowledge, no plan that we can adopt appears, in many instances, to exert much influence over its progress. The following rules may, nevertheless, be laid down:—

The patient should at first be bled two or three times, according to the strength of his constitution and the state of his general health; purgatives may also be given with advantage, and repeated once or twice. Then use the collyrium, containing the nitrate of silver, for six or eight days, applying at the same time the nitrate of silver ointment, if the free margin of the eyelid is inflamed. Should no amelioration take place during this period, it is useless to expect any benefit from the nitrate of silver; you must, therefore, give the other remedies we have examined a trial. If the various collyria and ointments fail to make any impression on the disease, you must, as a last resource, resort to cauterization with the solid nitrate of silver, the sulphate of copper, or the sulphate of iron.

*Purulent Blepharitis of new-born Children.*

Although by far the most serious of the different forms of inflammation we are examining, purulent blepharitis is the least refractory to the action of therapeutic agents. Leeches, emollient poultices, blisters, purgatives, &c., have been much extolled against this malady. They are undoubtedly valuable remedies, but as accessories only. Here, again, topical applications, aided by attention to diet, and the rules of hygiene, are alone to be depended upon for a radical cure, at least in the great majority of cases. Among these, the collyrium with the nitrate of silver or the salt in substance, when it is possible to cauterize directly the inflamed surface, have seemed to me more especially worthy of confidence. Sometimes the palpebræ are so much swollen that it is impossible to separate them sufficiently to employ the solution in the usual manner. In this case you may apply the remedy by means of a small syringe, the extremity of which is introduced between the palpebræ, near the outer angle of the eye. In every case, however, where it is practicable, the internal surface of the eyelids should be exposed, and the nitrate of silver lightly drawn across. Cauterization, practised in the above manner, has proved, in my practice, the most successful plan of treatment.

In the chronic stage, methodical compression, in addition to the means I have already mentioned, has often proved extremely serviceable. I need scarcely add, that extreme cleanliness is indispensable; and that in all cases, when it is practicable, the patients should be removed to a pure, healthy atmosphere, if the one they are living in does not present the desired conditions.

*Ciliary Blepharitis.*

I have but a few words to say respecting the treatment of ciliary blepharitis. It is the same, in nearly every respect, as that of the granular form of inflammation. The ointments most serviceable in the one, will also be found most serviceable in the other. Thus I have often subdued this affection with the nitrate of silver ointment, as also with those of Janin, Desault, and Regent. I must again remind you, that it is more especially during the first stage of this disease that the treatment you make use of, whatever it may be, is likely to succeed.

---

*On the Effects of Lesion of the Trunk of the Ganglionic System of Nerves in the Neck upon the Eyeball and its Appendages.* By JOHN REID, M. D.—In a former communication in this Journal I stated that I had frequently verified the observations of Petit and others, that when the *vagus* is injured in the neck in those animals in which the sympathetic is combined with the *vagus*, as in the dog,—that the *conjunctiva* becomes inflamed, the pupil contracts, and the eyelids are somewhat more closely approximated to each other. At that time I suggested that the contracted pupil and partially closed eyelids might probably depend upon the impatience of light which sometimes accompanies inflammation of the *conjunctiva*. I have since attended more carefully to this subject, and during the last summer satisfied myself that the contraction of the pupil, the projection of the cartilaginous membrane, or third eyelid, situated at the inner angle of the eye over the cornea, and the partial approximation of the eyelids to each other, take place immediately after the injury of the sympathetic, and before the inflammation of the *conjunctiva* presents itself, and that they continue after it has disappeared. I shall here detail a few of the experiments which I have made in elucidation of this point.

*Exp.* I. The *nervus vagus* and sympathetic were cut on the left side in a small terrier, and instantly the pupil of the eye became considerably diminished in size, and the cartilaginous membrane at the inner angle of the eye was forced over the internal margin of the *cornea.* A quarter of an hour after the division of the nerves, the *conjunctiva* of the left eye appeared more vascular than that of the right, and the *cornea* was perhaps a little dimmer. There was also some increased secretion of tears from the left eye, and the condition of the pupil and third eyelid remained as before.—Twenty-four hours. The *conjunctiva* of left eye was vascular and covered with a thick tenacious mucus, and the appearance of the *iris* and the third eyelid was the same as immediately after the division of the nerves. Some slight vascularity of *conjunctiva* of right eye, and some increased secretion of mucus, but there was no apparent change upon the pupil and third eyelid.—Third day. The *cornea* of left eye was dim, and the *conjunctiva* very vascular, and covered with much tenacious mucus. The *conjunctiva* of right eye was somewhat vascular, and covered with a small quantity of mucus.—Seventh day. The *cornea* of left eye was less dim, and there was less inflammation of *conjunctiva*, but it was still covered by a considerable quantity of puriform mucus. The pupil was still contracted, and the cartilaginous membrane projected over the *cornea.* The animal was now made the subject of another experiment. In this and in several other experiments which I have made, with a similar view, I was assisted by my friend Dr. Staberoh, of Berlin.

*Exp.* II. The *vagus* and sympathetic were cut across on one side, and a portion removed in a middle-sized cocker dog, after the exact similarity of the two eyes had been ascertained. Immediately after the section of the nerves, the pupil became contracted, the third eyelid projected over the inner edge of the *cornea*, the eyeball was apparently placed deeper in the socket, and rolled inwards, and the eyelids partially closed. At this time there was no perceptible redness of the *conjunctiva.* Ten minutes after the section of the nerves the pupil and eyelids remained in the same state, and there was no vascularity of the *conjunctiva.*—Twenty-four hours. The *conjunctiva* of the side operated upon was very vascular, with increased secretion of tears; the *cornea* was, however, clear, and there was no inflammation observable in the deeper parts of the eyeball. The opposite eye had undergone no change from the natural condition.—Forty-eight hours. No perceptible change since yesterday.—Fifth day. Rather less vascularity of the *conjunctiva* of the side operated upon, and it was partially covered with some puriform mucus. The other appearances remained as before.—Sixth day. Less vascularity of *conjunctiva*, no other perceptible change.—Eighth day. Vascularity of *conjunctiva* nearly gone, the pupil is apparently less contracted, and the third eyelid projects decidedly less over the *cornea.*—Tenth day. There are still some remains of conjunctival inflammation. The pupil is still very perceptibly smaller than that of the sound eye; the cartilaginous membrane projects less over the *cornea*, and the eyelids are less approximated, though evidently closer to each other than in the sound eye.—Third week. Scarcely any traces of redness on the *conjunctiva*; the third eyelid still projects forwards, but it does not now encroach on the *cornea;* other appearances remain as before.—Five weeks. Redness of *conjunctiva* entirely gone, but other appearances remain unchanged.—Two months. The pupil of the eye of the side operated on is still very decidedly less than that of the opposite side, and the eyelids are evidently somewhat more approximated. The pupil was not, however, motionless; but it has continued to contract and dilate when exposed to a stronger or feebler light. The animal was now killed by a dose of prussic acid, and it was observed that while expiring, the pupils of both eyes were much dilated, and became of equal size.

*Exp.* III. The left superior cervical ganglion of the sympathetic was removed in a dog in which the common carotid of that side had been previously secured to prevent hæmorrhage. Great care was taken to avoid injury of the *nervus vagus.* The lower half of the ganglion was at first only removed, and this was immediately followed by contraction of the pupil, the projection of the third eyelid over the inner edge of the *cornea*, and the other appearances remarked in the previous experiment. One minute after this the whole of the ganglion was removed without any apparent increase of the effects previously produced.—Twenty-four hours. Scarcely any increased redness of left *conjunctiva*, but there is some slight increased secretion of mucus. Pupil as yesterday. The eye remained nearly in the same state during the fortnight it was allowed to live. It ate freely, was quite active, and never showed any tendency to stupor.

In the dog, as I have already remarked, it is impossible to cut the *nervus vagus* in the middle of the neck without also dividing the trunk of the sympathetic. In the cat, however, though these two nerves lie in the same sheath, yet by a little care they can be easily separated from each other opposite the thyroid cartilage; and in the rabbit they have nearly the same relation to each other as in the human species. The cat and the rabbit consequently furnish an opportunity of experimenting upon these nerves singly.

*Exp.* IV. The sheath of the right carotid was exposed high in the neck in a kitten, and the sympathetic was then cautiously separated from the *vagus* without injuring the latter. When the sympathetic was compressed with a moderate force, the right pupil began to contract gradually, and became much smaller than that of the left eye; and it again resumed its former size on removing the pressure.* A portion of the right

* This experiment of producing contraction of the pupil by compressing the trunk of the sympathetic in the neck, I have repeated with success in other cases not detailed here.

sympathetic was now removed, and the pupil again contracted slowly, and remained permanently smaller than that of the left eye; the cartilaginous membrane was pushed considerably over the anterior surface of the cornea, and the eylids were partially approximated to each other.—Twenty-four hours. No distinct redness of *conjunctiva.* Condition of right eye the same as yesterday, and the pupil, though constantly smaller than the left, contracts and dilates within certain limits on exposure to a stronger or feebler light. The animal was lively. It died seven days after the division of the nerve. At the time of its death no distinct redness of the *conjunctiva* had presented itself, and the eye had the same appearance as the day after the operation.

*Exp.* V. The left sympathetic was cautiously separated from the *vagus* in the neck of a full-grown cat, and cut across. The iris immediately contracted slowly, and gradually, and soon presented a marked contrast to the right pupil, and the third eyelid was pushed over the inner surface of the *cornea.* The right *vagus* was now exposed, separated from the sympathetic, and divided without injuring the latter. This was followed by no change upon the right eye. The right sympathetic was now divided, and the same phenomena presented themselves as in the left eye. Dr. Alison was present at this experiment. No distinct redness afterwards presented itself in the *conjunctiva* of either eye. When the animal was killed three weeks after division of the nerves, the cartilaginous membranes at the inner angles of the eye, though more distinctly visible than usual, did not project over the *cornea;* the pupils were exactly similar, and appeared to have nearly recovered their usual size. As in this experiment, however, both sympathetics had been cut, we could not judge of the effects of the operation upon the pupil as in the cases where the sympathetic was divided on one side only, for then the sound eye served as a standard of comparison.

*Exp.* VI. I removed a portion of the right sympathetic from the neck of a full-grown cat in the presence of Dr. Monro and Mr. Mackenzie. On cutting the nerve across, the pupil was instantly seen to contract slowly and gradually, and soon presented a marked contrast with that of the opposite side. The cartilaginous membrane at the same time gradually encroached upon the surface of the inner part of the *cornea*, the eyeball appeared deeper, and the eyelids more approximated than those of the opposite eye. A month after section of the nerve, the size of the pupil still presented a very striking difference from that of the left eye; and the cartilaginous membrane projected more than that of the opposite side, though it no longer encroached upon the *cornea.* The animal never lost its activity.*

*Exp.* VII. In one rabbit the trunk of the sympathetic was first divided on one side of the neck, and a portion removed; and a few days after, the same operation was repeated on the opposite side. In other seven rabbits the superior ganglion of the sympathetic or a large portion of the trunk of the nerve was removed on one side without tying or injuring any of the large blood-vessels or any other nerve, and in two of these the same operation was repeated on the opposite side. In one of these only was there any change observed upon the iris, and no decided increased redness of the *conjunctiva* presented itself. In one of these animals it was remarked that the eyelids of the side on which the superior ganglion of the sympathetic had been removed, were less apart than on the opposite eye; but whether this was the effect of the removal of the ganglion, or of some slight injury received during the operation, we cannot at present pretend to determine. From these experiments it would appear that in rabbits the superior ganglion of the sympathetic, and a considerable portion of the trunk of that nerve as it lies in the neck, may be generally removed without effecting any change upon the iris; while the compression or section of the trunk of the sympathetic in the neck in dogs and cats is instantly followed by contraction of the pupil, the forcing of the cartilaginous membrane over the inner part of the anterior surface of the eyeball, the retraction of the eyeball deeper into the socket, and a slight approximation of the eyelids. In dogs this also is followed,—sometimes after a very few minutes, but generally after a longer interval,—by inflammation of the *conjunctiva*, which is occasionally so severe that this membrane presents an almost uniform redness, and is covered by puriform mucus, and the cornea becomes dim. As far as I have been able to observe this, inflammation is confined to the *conjunctiva.* To judge from the limited number of experiments which I have yet made upon cats and rabbits, the inflammation of the *conjunctiva* in the former is trifling, if present at all; and in the latter it is entirely absent. I was at first inclined to believe that the outward projection of the third eyelid, for in the dog and cat it has no muscles attached to it, was dependent upon the rolling inwards of the eyeball; but subsequent observations have nearly satisfied me that this depends upon the *retrahens oculi* muscle drawing the eyeball deeper into the orbit, by which the fat is pressed forwards, and the third eyelid pushed over the anterior surface of the eyeball. This would also explain the approximation of the eyelids. I find it impossible at present to give any thing like a plausible explanation of the effects of injury of the sympathetic upon the eyeball and its appendages, and the cause of their dissimilarity in different animals. It is evident, however, that this is to be sought for in the connection of the branches of the sympathetic with the encephalic nerves of the orbit, and especially with the sixth pair, and those branches forming the ciliary nerves. I intend at my earliest opportunity to endeavour, by extensive minute dissections of the ascending branches of the superior

* In a cat on which I assisted Mr. Little to repeat this experiment, the pupil was nearly natural a month after a portion of the sympathetic and *par vagum* on one side were rem ved.

sympathetic ganglion in various Mammalia, to give some probable solution of this question. We may then be able to judge whether an injury of the cervical portion of the sympathetic in man, such as may possibly occur in certain diseases and operations on the neck, would be followed by contractions of the iris and inflammation of the *conjunctiva.* In a case described in the Medical Gazette,* where the right carotid, the vagus and surrounding parts are described as being enveloped in a large morbid tumour, and where, consequently, the sympathetic could hardly be supposed to escape, the pupil of that side is described as becoming smaller during the course of the disease.

This contracted state of the pupil, consequent upon lesion of the sympathetic in the neck, is not noticed in the experiments of Cruickshank,† Arnemann,‡ Mayer of Bonn,§ and Brachet,|| though all these authors describe its effects upon the *conjunctiva.* Indeed, Arnemann expressly states, "that he has not observed the changes upon the pupil which Molinelli has remarked after ligature of the eighth nerve;"¶ and Brachet, in relating his first experiment, where the trunk of the sympathetic was divided in the neck of the dog for the purpose of observing its effects on the eye, makes the following observation:—"L'iris n'en a reçu aucune influence marquée; il a continué de se contracter suivant la vivacité de la lumière: cette remarque ayant été commune à toutes les experiences, je ne la reproduirai pas." Petit** remarked this contraction of the pupil in some of his experiments; but his observations would lead us to believe that it was a subsequent and not an immediate effect, and could not enable us to decide whether or not it was dependent upon the inflammation of the *conjunctiva.* In Experiment I. of the second series of his two sets of experiments,†† the right vagus was cut on the 18th September; and on the 19th, he remarked, among other changes on the eye of that side, that the pupil was less. The left vagus of the same animal was afterwards divided, (Experiment II. p. 11,) and in a quarter of an hour he observed the cornea flattened, and the pupil contracted. In Experiment III. and IV. he observed the pupil to be contracted one hour after section of the nerves. In Experiment V. he made this curious observation, that when the nerves were cut on both sides, dilatation of the pupil followed, and more in the right eye than in the left. This animal, it is worthy of remark, suffered much from dyspnœa, and died in twelve hours. In the numerous experiments which I have made upon the effects of section of the vagi, I never observed any thing similar to this; on the contrary, the pupils always became contracted.

Molinleli* relates five experiments upon dogs, in which he watched the effects of ligature of the vagus and sympathetic upon the eye. In one experiment he informs us, (p. 281,) that a little after the left vagus was tied, the *conjunctiva* of the left eye became red, and the cartilaginous membrane at the inner angle of the eye projected over the cornea. On the seventeenth day after the operation, he observed that the pupil of that eye was diminished in size. In Experiment III. (284) the left vagus was tied with a double ligature on the 14th January; and on the 30th it was remarked that the pupil of the left eye was twice as small as the right, and that the eyeball seemed depressed. In Experiment IV. he mentions a change of colour in the iris; but there is nothing said about the diminution of the pupil. Dupuy† in two of his experiments mentions this contraction of the pupil. In one experiment (Oper. cit. Premier Fait, p. 343,) he remarked that the pupil became contracted immediately after the superior ganglion of the sympathetic had been extirpated. His words are these:—"Aussitôt après l'operation, l'œil de ce côté parut plus enfoncé dans l'orbite, les paupières étaient tumifiées, la membrane clignotante se portait en avant du globe occulaire la pupille se ressera." In a subsequent experiment, (Troisième Fait, p. 347,) the operation was performed on the 26th April; and on the 10th May he observed that the pupil was contracted. Though I have occasionally observed very severe inflammation of the *conjunctiva* follow this operation in dogs, yet I have never seen it proceed to the disorganization of the eyeball. In one of Arnemann's experiments it appears to have produced ulceration of the cornea.‡ In two of Mayer's experiments upon rabbits, where the vagus and sympathetic were not only enclosed in ligatures, but the common carotid was also tied, inflammation of the cornea occurred, and this in one case was followed by ulceration, and in the other by staphyloma, and the effusion of a layer of lymph upon the anterior surface of the iris, obliterating the pupil.§ Brachet relates several experiments to show that injury of the sympathetic or destruction of its superior cervical ganglion is attended by great vascular congestion of the anterior and middle lobes of the brain, producing drowsiness and stupor.|| The experiments which I have made on this point do not

* September 29, 1838, p. 16.

† Medical Facts and Observations, Vol. vii. p. 136, or Phil. Trans., 1795, Part I.

‡ Versuche über die Regeneration der Nerven, S. 69, 85–6–7–9, 94–6–7–9, 102. Göttingen, 1787.

§ Journal der Chirurgie von Gräefe und Walther, Zehnter Band, S. 418.

|| Recherches Experimentales sur les Fonction du Systeme Nerveux Ganglionaire, Chap. ix. Experiment 150–1–2, &c.

¶ Oper. cit. s. 96.

** Memoire dans lequel il est demontré que les Nerfs Intercostaux fournissent des rameaux qui portent les esprits dans les yeux. Histoire de l'Academie Royale, 1727.

†† Oper. cit. p. 10.

* Comment. Bononiensi, Tom. iii. 1755, p. 280.

† Journal de Médecine, Chirurgie, &c., December, 1816, Tome xxxvii., p. 340.

‡ Oper. cit. Acht und Sechzigster Versuch, S. 69.

§ Oper. cit. Experiment 17 and 18.

|| Oper. cit. Experiment 155–7–9 and 160.

by any means confirm those of M. Brachet. As the fact, if correct, is one of great importance, and ought to be very carefully investigated, I shall reserve the consideration of this and some of the other questions connected with the lesion of the cervical portion of the sympathetic, and more especially the length of time the contracted state of the iris continues after a portion of the nerve has been removed, until a future opportunity.—*Edinburgh Med. and Snrg. Journ.*

---

*Case of Dislocation of the Femur into the Ischiatic Notch, with Fracture. The Dislocation reduced after the lapse of six weeks.* By CHARLES THORNHILL, Esq.—Samuel Sheldon, æt. about 40, of short stature, but strong muscular make, was at work as usual at Tibbington Colliery, on the morning of the 6th of February, when a large fragment from the excavated roof of the pit became disengaged, and fell upon him. The portion that struck him was supposed to be about five or six hundred weight, which, coming in contact with the lower part of his back and right hip, while he was in the act of leaning forward, spent itself out upon the thigh of the same side. He was nearly stunned by the violence of the shock, but was immediately drawn up out of the shaft, and conveyed in a chair to his residence—a distance from the colliery of between two and three miles.

Mr. Roberts, the surgical attendant of the colliery, having at the time some pressing engagement, sent a note to request that I would take the charge of the case. Accordingly, in somewhat more than two hours after the occurrence of the accident, my brother saw the patient, and, on examination, discovered a slightly oblique fracture of the right femur, at the upper third of the bone. Several severe scratches and bruises were visible about the loins and pelvis, and it was evident that the hip also had sustained some considerable injury; but in consequence of the great tumefaction of the soft parts, it was impossible to ascertain accurately the extent of the mischief. The fracture being reduced, the limb was dressed and secured in the usual manner, and afterwards placed in the straight position. I had not an opportunity of seeing the patient until a week or more subsequently, when the swelling around the hip had so far subsided as to enable me clearly to distinguish a dislocation of the head of the femur from its appointed acetabulum. No attempt, however, could at present be made for the accomplishment of reduction, on account of the want of necessary firmness and stability in the shaft of the bone; and it was resolved to wait patiently until reunion between its fractured extremities should become firmly established, and then to adopt such means as might be deemed most desirable to meet the urgency of the case. Every thing continued to progress in a very satisfactory manner, but notwithstanding all our entreaties, the patient could not be prevailed upon to remain in bed longer than a month.

After the expiration of the fifth week, it was proposed to endeavour to effect reduction; but at the suggestion of a neighbouring medical friend, who thought that the restorative powers of the system had hardly yet contributed sufficient strength to the limb as to enable it to offer resistance to the mechanical force which would necessarily be applied, the operation was deferred for another week. The day was at length fixed for the trial of our means; and Mr. Roberts, of Dudley, and Mr. Lees, of Wednesbury, were kind enough to render their valuable assistance on the occasion. We assembled for that purpose, therefore, on Wednesday morning, the 20th of March, exactly six weeks from the period at which the accident had happened.

The patient was directed to undress, in order that the limb might be minutely examined by the gentlemen present. When he stood upright, and allowed the left leg to support the weight of the body, the unsound limb was observed to be about one inch and a half shorter than the other; and from the great trochanter being felt rather in the rear of the acetabulum, and from the inclination inwards of the knee and the foot together, with the pointing of the great toe towards the ball of that on the opposite side, it was at once pronounced to be a luxation into the ischiatic notch. But, independently of these diagnostic marks, the emaciation that had supervened upon the accident was so great, that the head itself of the femur could be felt lying in that foramen. The muscles around the joint seemed remarkably flaccid, and as there was a greater power of motion than is ordinarily witnessed, it was anticipated that we should have but little difficulty in effecting reduction. The sequel will show whether or not our expectations were realized. A door, with a blanket thrown thereon, was placed upon the bed, and two staples having been driven into the opposite walls, the patient was instructed to recline upon the door on his left side, with the right shoulder drawn one-third backwards. This latter precaution was deemed necessary, in order to prevent his being drawn forwards upon his face by the first application of the extension. A well-padded band was then passed between the thighs, close upon the perinæum, and this was affixed to the staple at the back of the patient, when, the pulleys being secured to the opposite staple, as well as to the rings of the knee-belt which had been previously applied, extension was made in the usual manner in a line directly across the middle of the uninjured thigh. After this had been steadily and perseveringly employed for upwards of half an hour, without occasioning much relaxation in the muscles, it was thought advisable to administer the antimonii potassio-tartras, in doses of a grain, repeated at intervals of a few minutes, until nausea should be fully obtained. When three or four doses had been given, the head of the bone began to advance towards the acetabulum, and I could distinctly feel it disengaging itself from its recently formed attachments. At this stage of the operation, my brother having passed a strong band under the upper part of the thigh, got upon the door, and fastening the loop of the band over his own shoulders at the same time that he pressed firmly with his hands upon the crista of the ilium, endeavoured to lift up the head of the bone, while Mr. Roberts forcibly rotated the limb outwards, by grasping it both at the knee and ankle joints. Whilst this was going on, one of the straps that secured the band between the thighs suddenly gave way; but as we had another band in readiness, it was applied over the other without loss of time, and without much reducing the amount of extension already obtained. As soon as this was properly adjusted, similar efforts were again resorted to, and continued for some time without success. By this, he had taken about ten grains of the tartar emetic, and the system began to be somewhat under its influence, as was manifested by the increasing pallor of the skin, and the comparative looseness and tremor of the muscles in general; but while we were employed in augmenting the extension, another difficulty presented itself, in consequence of the knee-strap gliding over the surface of the patella, although the precaution had been adopted throughout to keep the limb at right angles. This was replaced as speedily as possible, and our efforts were renewed as assiduously as before. After the patient had taken the twelfth dose of the potassio-tartrate, he became very restless, and it was evident that nausea had been duly produced. He now implored us most earnestly to desist from the further use of means; and as extension had been carried as far as was justifiable, it was resolved, before we abandoned the case as hopeless, that another and a last attempt should be made. For this purpose, my brother endeavoured once more to elevate the bone, while Mr. Roberts performed rotation outwards, as already described; and after these efforts had continued for two or three minutes, the head of the femur was forced into its socket with an audible crash, the poor fellow having been under treatment for an hour and three quarters. On relaxing the pulleys and disengaging the apparatus, the limb was found to be of nearly equal length with the other; but the difference that existed might be wholly attributable to the effects of the fracture.

The empl. thuris comp., spread upon linen, was applied over the joint, and the bandage ordinarily used in such cases was passed round the waist, and closely bound over the unsound hip. The patient was then placed in bed, and ordered to remain there for the space of a fortnight. Before the expiration of this time, however, he got up, and went out of doors upon crutches, though as yet he was unable to bear weight upon the leg.

About this period he came up to my surgery to be dressed. On examining the hip, there was considerable enlargement and disfigurement, arising from the thickened state of the muscles and integuments, and also from a large mass of callus which had been deposited about the joint; and to so great an extent had coagulable lymph been thrown out, that it was extremely difficult to trace the characteristic prominences of the bony structures. Indeed, in no case that had ever fallen under my observation, do I remember having previously witnessed such an immense deposit in the neighbourhood of the acetabulum; notwithstanding, the different motions of the limb, as far as they could be performed, were perfectly natural, and the man seemed highly pleased with the progress he had already made. The mercurial plaster was now substituted for the empl. thuris comp., and this was renewed every week for about a month or five weeks. But as absorption had taken place only to a trifling extent, and as the motion of the limb continued to be in a measure impeded by the callous mass, the size of a nutmeg of the iodine ointment, in the following proportions, was directed to be rubbed every night around the joint:—

℞ Potassa Hydriod.; Tinct. Iodinæ, āā ʒj.;

Ung. Cetacei, ℥j. M.

On the 10th of May he walked on crutches to Dudley, a distance of five miles, where he was seen by Mr. Roberts, and also Mr. Badley, who concurred in the propriety of continuing the iodine preparation for a few weeks longer.

At the time of the present report, (May 29th,) he represents himself as rapidly gaining strength and power of mobility in the joint. He is able to bear his entire weight on the injured side, and he talks of substituting a stick for the use of the crutches. There has been considerable absorption of the morbid deposit, though it is still sufficient to occasion a slight disfigurement about the hip. Much of this callous mass, it is to be feared, will remain permanently; but this will not be of any great consequence, as even now it does not appear to interfere materially with the motions of the limb. These have already become very extensive, but some months must necessarily elapse before they are perfectly restored to their pristine condition.—*London Medical Gazette.*

---

*On the Changes in the Blood in Inflammatory and Typhoid Diseases.*—Andral has with great justice remarked in his Precis d'Anatomie Pathologique, that "the consequence of every alteration of the solids must necessarily be an alteration of the blood, just as every modification of the blood must be followed by some modification of the solids."

Admitting then the truth of this important pathological axiom, it is evident that in any disease, *tant soit peu pronouncée*, the organ, which appears to be affected, is not the only one that is disordered, since the whole economy must necessarily suffer from the disturbance of any of its parts. This doctrine has of late years been gradually gaining ground, and very few even of the followers of Broussais refuse now to admit the existence of an alteration in the blood itself as a necessary element—superadded to, if not actually induced by, the *gastro-enterite*—in the ætiology of all typhoid fevers. In some diseases, such as scurvy, cholera, glanders, eruptive fevers, &c., the *hæmatic* change is the primary and essential phenomenon, to which all their symptoms are referable, as to their exciting cause. Even in respect to the extensive class of the phlegmasiæ, the opinion held by Sydenham, and most of the older physicians,—"that they all proceed from inflammation of the blood itself, causing a deposition of the morbific matter upon the lungs, brain, joints, or other organ most affected,"—has been adopted by some able cotemporary physicians, who hold that the disease in the solids is always preceded by that of the circulating fluids. Other physicians maintain the converse of this proposition, and contend that the inflammatory state of the blood is always preceded by the phlogosis of the solids. The question is still undecided. But, be this as it may, no one will hesitate to admit that the state of the circulating fluid affords one of the surest and most constant indications of the existence of any active inflammation. Let us now endeavour to ascertain, more exactly than has been done hitherto, the nature of those changes which the blood exhibits during this morbid process.

In perfect health, the globules of the blood seem to have a much more feeble attraction for each other, than when the inflammatory or phlogistic diathesis is present. Compare the blood drawn from a person in health with that from a patient affected with an active phlegmasia. In the former, the clot or coagulum is usually more bulky, and is at the same time much less firm or resisting to the impression of the finger, than in the latter. Inflammation seems to modify all the characters of the blood, by augmenting the force of attraction between its molecules; the fibrinous globules, which in a healthy state are not very closely drawn together, but are separated somewhat by the serosity, and enveloped with the colouring matter, are then powerfully attracted towards each other; and thus the coagulum, from the squeezing out, as it were, from its pores, of all the serum and much of the colouring matter, acquires that firm, dense, and puckered-on-the-surface appearance, which is so conspicuous in highly inflamed blood.

The lower surface of such a coagulum always presents, it is well known, a layer of soft, sometimes almost gelatinous, and deep red coloured bloody matter; this is owing to the settling down of the greater quantity of the colouring globules to the bottom. Hence it will be generally found that the clot of inflamed blood will be more resisting on its upper surface, and less resisting on its lower one, than healthy blood. To this is to be added, that there is usually a larger quantity of serum in the former than in the latter, in consequence of all the serosity having been, so to speak, squeezed out from the interstices of the fibrinous globules in the one case. The character of the clot, therefore, affords most valuable signs to direct the intelligent physician in his diagnosis and treatment of many diseases.

Whenever this is unusually firm, and tough or resisting to the impression of the finger, whether there be any buffy coat on its surface or not, we may be assured that an inflammatory condition of the system is present, and that our patient's case requires an antiphlogistic treatment. This simple rule, therefore, may afford a useful guide to the practitioner in treating certain cases of inflammation, which are accompanied with symptoms of such prostration as might seem quite to contra-indicate the employment of any lowering remedies. We should trust less to the mere existence, or not, of a buffy coat on the surface, than to the general consistence or firmness of the entire coagulum. The more active the inflammation, the firmer and tougher will the clot be found, and *vice versa.* We are of opinion that M. Piorry, in his ingenious discourse on *hæmitis* or inflammation of the blood, is in error when he insinuates that this state is invariably indicated by the formation of a buffy coat on its surface, after it has been allowed to rest for some time. According to our view of the case, the general increase in the aggregation of the particles of the clot is a safer and more exact test of the condition referred to.

This increased force of aggregation of the fibrinous globules would seem, in some cases, to overcome during life its antagonist power, which keeps the blood fluid in the vessels. In this manner the formation of coagula within the cavities of the heart—the presence of which during the progress of some cases of pleuropneumonia has been repeatedly discovered, by means of auscultation, by M. Bouillaud—is, often at least, to be accounted for. M. Raciborski states that he has on several occasions verified the truth of this opinion, and that he has observed that, when in such cases the plasticity of the blood is diminished by bloodletting, &c., the abnormal sounds of the heart gradually abate and disappear.

So much for the usual state of the blood in *inflammatory* or *phlogistic diseases.* We now proceed to examine the condition of this vital fluid in *typhoid fevers.*

M. Raciborski has kept detailed notes of the state of the blood drawn in cases of typhoid fever, 111 times by venesection, and 68 times by the cupping glasses. In 46 of the 111 bleedings from the arm, the blood exhibited no buffy coat, (*couenne,*) or only a few soft and semi-transparent patches of it. The coagula, in all these cases, were more or less soft, but presented considerable variety in point of consistence, some being firm enough to bear half of their own weight, while others gave way to the pressure of the finger in attempting to take them up. The serum was always in small quantity, and somewhat opaque or troubled; it was never so clear as the serum of blood drawn during an inflammatory disease.

In 18 of other 27 venesections, the blood drawn did exhibit a buffy surface; but in almost all these cases, the buff was thin, semi-transparent, and gelatinous.

We now come to state the appearance of the blood in the remaining cases, in all of which the typhoid fever was complicated with symptoms denoting inflammation of the lungs, throat, head, &c. In five out of twelve cases, in which the fever was accompanied with bronchitis, the blood drawn presented no buffy coat, and its coagulum was soft, and easily lacerable. In all these cases the inflammatory affection was slight and limited.

In other 24 venesections, practised on seven patients, the blood exhibited a buffy coat 17 times; but in no case was it thick, tough, and leathery, as we see it in simple active inflammation.

From all his observations, M. Raciborski concludes that, in typhoid fevers, even when they are complicated with an inflammatory affection of some of the viscera, the blood very rarely exhibits a genuine buffy coat, such as we observe in *inflammations franches.*

We have already stated that the consistence of the coagulum, subjacent to the buffy coat, is always inversely proportionate to the thickness and toughness of this coat; the thicker and denser the buffy coat is, the softer and more yielding is the subjacent coagulum. The reason of this phenomenon is obvious: when the buffy coat is very thick, the greater part of the fibrinous portion of the blood has separated from the colouring globules, and these latter, in consequence of their greater specific gravity, sink to the bottom.

Now, the blood drawn in typhoid fever never exhibits so thick and tough a buff on the surface of the clot, nor so soft and gelatinous a consistence at its base, as that drawn in an active inflammation. The transparency of the serum, being in general proportionate to the firmness of the coagulum, is seldom or never so great in the former as in the latter case.

These various phenomena are all attributable to the increased force of aggregation or attraction between the molecules of the blood in the one case, and to the diminished force of the same power in the other.—*Med. Chirurg. Rev., from Gazette Medicale.*

---

### PROCEEDINGS OF THE PATHOLOGICAL SOCIETY OF DUBLIN.

*Fistulous Opening upon each side of the Neck, communicating with the Œsophagus.*—Mr. Cusack presented the œsophagus taken from the body of a man who had been affected with venereal, and had used mercury irregularly. He was admitted into Stevens' Hospital in a broken down state of health; there was a remarkable fulness on one side of the neck, with two tumours, one on each side of the mesial line, above which were ulcers, apparently of a venereal character; another existed near the clavicle. The patient improved under the use of sarsaparilla; a small quantity of mercury was then exhibited; and subsequently it was found that the greater part of his food and drink escaped through the fistula in the neck. The patient died suddenly, with symptoms of peritonitis from perforation. A perforating ulcer was found in the small intestines; the ulcers in the neck were found to communicate with the œsophagus by two openings, around which the appearances of disease in the tube were much slighter than could be expected in a case of the kind. Mr. Cusack, however, believed that the ulcerative process had begun in the œsophagus; the trachea was healthy. Mr. Cusack was not aware that any similar case was upon record. (*Museum, Park street.*)

2. *Condensation of the Cellular Tissue in Infants.*—Dr. Evory Kennedy said he had an opportunity of exhibiting a form of disease which had for a long time occupied the attention of the profession on the continent, but had not been the subject of much investigation in this country, probably from the circumstance of its being comparatively rare; it was known by the name of *condensation* (or *endurcipement*) of the cellular tissue, and was observed almost exclusively in new-born children. It consisted in a remarkable hardening of the whole or a portion of the cellular substance, generally commencing in the hands or feet, and gradually extending to the abdomen and face, accompanied by coldness and paleness of the skin. In some points it bears a resemblance to the phlegmasia alba dolens which affects females after delivery. It is considered by some to arise from cold, followed by obstruction of the transpiratory vessels, and infiltration into the cellular tissue of a gelatinous fluid. Others think that there is no infiltration, and that it is merely a condensation of the cellular tissue, without any extravasation. In some instances, but very rarely, it terminates in suppuration; in fact, this has been a disputed point among those who have examined the subject. The case, however, exhibited by Dr. Kennedy, would be sufficient to set the matter at rest, for a small purulent deposit existed in the cellular tissue under the abdominal integuments. Some look upon this disease as the consequence of syphilis; others, among whom is Breschet, think that it depends on a peculiar state of the circulation, connected with a patulous condition of the foramen ovale, and disease of the lung. Both opinions were supported to a certain extent by the case before him, for the child presented some of the phenomena observed in cases of venereal, whilst there was a highly developed state of one lung, with carnification of the other. It was an affection of a very obscure description, and required further investigation.

3. *Conversion of the false Membranes of Pleuritis into Tubercle.*—Dr. Stokes exhibited drawings illustrative of this pathological condition. The case was that of an individual aged about thirty; he was a soldier, and had undergone repeated punishments in this country, and also in the West Indies; he was imprisoned, when his health, which had been much broken before, gave way, and symptoms of pulmonary disease became developed; he was admitted into hospital with all the symptoms and signs of pneumo-thorax from fistula of the left lung, and softened tubercle in considerable quantity in both lungs; the perforation occurred six weeks before admission, and the patient lived three months from that period.

The sac of the empyema presented very different appearances when viewed on its interior and exterior aspect. Interiorly, the pleura was covered thickly with a deposition of a yellowish white lymphy matter, with a remarkably variegated aspect, and irregularly granular appearance; the deposition next the pleura was of a rose-red colour, and that was as it were speckled over thickly with white and opake masses of an irregular form; some roundish, others angular, some without any trace of vascularity, others deeply injected or almost ecchymosed. This appearance existed over the whole surface of the sac.

The lung and costal pleura being removed carefully, it was found that a vast number of flattened spheroidal masses, of a yellowish white colour, and perfectly defined, were seen shining through the pleura, when examined on its costal or posterior surface. Their diameter varied from that of a split pea to that of a millet seed. They did not exist on the posterior surface of the pleura, but were evidently incorporated with the false membrane on its interior surface. The vascularity of a number of these masses was obvious.

Dr. Stokes referred to the observations of Laennec and Hodgkin on this very rare pathological condition; he agreed with Laennec that in such instances the tubercular matter was not secreted by the pleura, but produced by a specific degeneration of the false membrane itself. (*Museum, Park street.*)

4. *Extensive Ossification of the false Membranes in Pleuritis, with Encysted Hydrothorax.*—Professor Harrison laid on the table the lungs of a man who had died with effusion into the left pleura, and congestion of both lungs. The right pleura exhibited evidences of having been formerly attacked with inflammation, and on its anterior face a vast plate of bone, of great density, was discovered. The lung was covered by cellular adhesions, having a fibrous structure, along the course of which the ossific process seemed to have proceeded, so as to resemble the appearances observed in the growth of the bones of the infant's skull; very long and sharp spiculæ diverged in every direction. In the lower part of the left pleura, about a pint of clear fluid was found encysted. The walls of the cavity were strong and polished, somewhat like those of an hydrocele. During life, dulness of sound and absence of respiration had been observed in this situation. (*Museum, Trinity College.*)—*Dublin Journal.*

---

*Glanders in the Human Subject.*—M. *Andral* communicated the following report at a recent meeting of the Royal Academy of Medicine:

An ostler, addicted to drinking, had for several months slept in a small stable, where there were usually some diseased horses, which it was his duty to attend to. Two of the horses were glandered, and have been within the last few weeks slaughtered at Montfaucon. Another ostler, who also slept in this stable, died very suddenly; but of what disease it is not known. Immediately after his death, the subject of the present report was taken ill, on the 11th of January.

The first symptoms of his illness were such as usually usher in an acute malady, viz., lassitude, pains in the limbs, headach, prostration, &c. This is the first stage, or that of the invasion of the disease. The second is characterized by an increase in the severity of the symptoms now mentioned, and by an eruption on the skin, which we shall now describe. (It was at this period of the disease that the patient was admitted into the Hôpital de la Charité.)

At first sight, the case was regarded as one of pustular erysipelas; the face and forehead being much swollen, puffy, red, and covered with papulæ. On further examination, however, it was found that similar pustules of a livid colour were scattered over the limbs, the cavity of the mouth, and the nasal passages: five or six abscesses co-existed with the eruption upon the upper and lower extremities. There was no outward discharge from the nostrils; but it was discovered that a bloody puriform matter flowed back from the nostrils into the throat. The breathing became gradually more and more distressed; coma supervened, and the patient rapidly sunk.

*Dissection.*—The following lesions, which present a striking resemblance to those in other five cases, which have been noticed in Paris within the last two years, were found by M. *Andral.*

The surface of the body exhibited numerous gangrenous ulcerations wherever any pustules had appeared. Several abscesses and extensive purulent sinuses were found in the muscles of the limbs; and these extended down, in some places, to the bones themselves. The mucous membrane of the intestinal canal was spotted with numerous petechiæ and patches of ecchymosis; that of the nasal passages was ulcerated, gangrenous, and infiltrated with purulent matter; the *ossa spongiosa* were denuded at several points. The *velum palati* also was partially gangrenous and infiltrated: these morbid changes extended down along the pharynx and larynx. The lungs were affected with lobular pneumonia, as they usually are in such cases; and at the summit of the right upper lobe was an unhealthy fœtid abscess.

In conclusion, it may be stated that some of the matter from the nostrils and of the discharge from the pustules was inoculated upon an ass, and that the animal was speedily affected with the nasal flux peculiar to acute glanders.—*Medico-Chirurg. Review, from Memoires de l'Academie.*

---

*Blennorrhœa Urethræa, accompanying the Teething of Children.*—There is a case of this kind recorded in the Medicinische Zeitung for November, 1838, by Dr. Mehliss of Liebenwerda, which bears so strong a similitude to a case given by John Hunter in his Natural History of the Teeth, that we thought it worthy of insertion.

The child of a shoemaker, two years and a half old, became ill on the 21st February, apparently of catarrhal fever, for which my advice was sought on the 26th. The fever was high, but the cough slight; the abdomen tense and hard, the tongue coated, and the bowels costive; much thirst, but great dislike to food, and it complained of pain in passing water; the urine was turbid like whey, and deposited a whitish yellow sediment. On examining the virile member, I saw with astonishment that the prepuce was red, and that a puriform fluid issued from the orifice of the urethra, the lips of which were swollen and inflamed, just as is usual in common gonorrhœa. The parents of the child had not remarked this discharge, and did not know how long it had continued. The habits of life in which both parents and child lived did not admit of any suspicion being entertained of this being a venereal affection, and I was of opinion that this blennorrhœa might be caused by the irritation of worms in the intestinal canal. Consequently, I ordered aperient clysters, containing infusion of santon seeds to be thrown up, and also gently opening medicine to be administered, which caused abundant evacuations, but without any worms being discharged. After this, both fever and cough disappeared, the appetite again returned, and the discharge from the urethra diminished. On the 29th of February, the child's parents remarked that the right canine tooth in the upper jaw, the only one of the milk teeth which had been deficient, had just burst through the gum. Some days after this, all symptoms of disease disappeared, as well as the discharge from the urethra, and the child was as healthy as before.

In John Hunter's case, the child, two years old, was affected with pain and difficulty in passing urine, and genuine pus issued from the urethra. Hunter thought that the child was infected with venereal poison, and suspected the nurse. However, he remarked one unusual circumstance in gonorrhœa, that the affection was sometimes less, sometimes disappeared altogether, and then returned. At last it was discovered, that with the appearance of each new tooth the affection came on, and this with so much regularity and certainty as to leave no doubt of its being merely a sympathetic inflammation.—*Dublin Journal.*

---

*Styptic Action of Creosote.*—Drs. *Muller* and *Reiter* have lately instituted a series of experiments for the purpose of ascertaining the styptic properties of creosote, when applied directly to a bleeding surface. The hæmorrhage from the division of the crural vein in dogs was found to be arrested by the application of a plug of cotton, which had been well moistened in the creosote.

In the case of divided or wounded arteries, it was necessary to keep up a certain degree of compression for some time, in order that the creosote might be able to act upon their parietes. Upon examining the cut arteries afterwards, they were always found to be quite closed or obliterated at the part, exhibiting outwardly an umbilical depression, which corresponded with a conical-shaped coagulum within: the coats of the vessel were usually inflamed for the extent of an inch or so.

The creosote was found to be a more decided and secure *hæmostatic* remedy than the far-famed aqua Binelli. *Creosoted water* sufficed to stop the bleeding from an oozing surface, when no large vessels had been divided. When creosote was injected into veins, the blood was found to be instantaneously coagulated.

Professor *Schneider*, of Munich, had recently an opportunity of using creosote as a styptic in one of his patients. An old man was subject to most profuse hæmorrhage from the mouth. He had lost several pounds of blood, and a variety of means had been ineffectually tried to arrest the hæmorrhage.

M. *Schneider* made him fill his mouth with water charged with creosote (*eau creosotée*;) after the third mouthful, the bleeding ceased, and did not afterwards return.—*Med. Chirur. Rev. from Schmidt's Jahrbucher.*

[An interesting case illustrating the styptic properties of creosote is reported by Dr. I. Parrish, in No. 32.—Eds.]

---

*Epilepsy cured by Acupuncture.*—Henry Mayer, a Swiss soldier, of good constitution, was affected with scabies in 1837, of which he was soon cured. Shortly after the patient was attacked by acute pain in the left shoulder, gradually extending to the arm, and which resisted every remedy employed. The pain eventually extended to the whole of the left side of the body. One evening, while traversing a long corridor, he was alarmed by one of his companions, and immediately seized with epileptic convulsions. The latter continued, and soon assumed the character of true epilepsy, the access returning every two or three days.

On his admission into the hospital the left lower extremity dragged along the ground, as if paralysed, and the patient's condition was extremely unfavourable. M. Gambarini having been present one day during an epileptic attack, conceived the idea of passing an acupuncture-needle into the region of the heart, and accordingly introduced one, to the depth of three inches, between the fifth and sixth ribs. The fit was immediately arrested. On the appearance of the next epileptic attack it was stopped in a similar manner, and the remedy had constantly the same beneficial effect.

The state of the patient gradually improved, and a complete cure was obtained. He remained in the hospital forty days afterwards, without experiencing a single attack.—*London Lancet, from French Lancet*, July, 1839.

---

*Lithotrity.*—M. Sanson, of the Hôtel Dieu, has recently undergone the operation of lithotrity with the happiest result. During the whole period of treatment M. Sanson was not compelled to forego his consultations for a single day.—*Lancet.*

